# Waffle production: influence of batter ingredients on sticking of fresh egg waffles at baking plates—Part I: effect of starch and sugar components

**DOI:** 10.1002/fsn3.424

**Published:** 2016-09-20

**Authors:** Regina Huber, Regine Schoenlechner

**Affiliations:** ^1^ University of Natural Resources and Life SciencesDepartment of Food Sciences and TechnologyViennaAustria

**Keywords:** Fresh egg waffle, starch, sticking, sugar, waffle batter

## Abstract

Fresh egg waffles are a sweet convenience product typically baked from eggs, water, sugar, flour, fat, leavening agents, emulsifiers, preservatives, and flavors. In industrial production, waffles are baked continuously in high amounts of up to 200 kg raw material per hour. Therefore, it is important that the waffles do not stick onto the baking plates, which can cause significant product loss and increased costs due to interruption of the baking process, required cleaning procedures, and restarting of the energy‐consuming start‐up phase. Sticking of waffles is greatly influenced not only by baking plate material, release agent, baking temperature, and time, but also by the batter ingredients. In this study, effects of different starches and sugar components were investigated. Within the selected starches, potato starch demonstrated the highest effects on increasing waffle stability and releasing properties compared to wheat and lupine flour (less than 7% sticking waffles). Rice flour performed worst, with almost 50% of sticking waffles. Most of these waffles were broken during take‐off, due to their crumbly texture. Within the sugar components, glycerine was better suitable than sorbitol and crystal sugar was superior compared to powdered sugar. They required less take‐off force. It could be demonstrated that waffles with increased stability and texture were those that showed the least number of sticking waffles, thus the main aim of batter ingredients was to improve waffle quality. Waffle quality was influenced by batter parameters, significant correlations could be found, for example, a positive correlation between pH‐ and L‐value, negative correlations between pH‐ and a‐value, or density and aw‐value. This resulted in significant correlations with take‐off‐force, which was correlated with L*‐ and b*‐value (negative) and positive to a*‐value. Sticking behavior was strongly associated with b*‐value (positive) and to a*‐value (negative).

## 1 Introduction

Fresh egg waffles (subsequently called waffles throughout this manuscript) are a sweet convenience product with a soft texture, similar to a cake. Typical ingredients for waffles are eggs, water, sugar and sugar substitutes, flour and starch, fat, leavening agents, emulsifiers, preservatives, and flavors (Tiefenbacher, 2009). In industrial scale, waffles are baked at 140–180°C for 110 and 180 s, depending on thickness of waffle and batter type (Ashokkumar & Adler‐Nissen, 2011; Tiefenbacher, 2003). Important is that the waffles are fully baked but not burnt, and that they do not stick onto the baking plates. If a waffle sticks to the baking plate, completely or partly (when it is torn during take‐off due to insufficient waffle stability), it must be removed by hand and the baking plate must be cleaned. This interrupts continuous waffle baking in the industry, which can cause significant product loss and increased costs and thus has to be kept to an absolute minimum (Chavan, Sandeep, Basu, & Bhatt, 2016; Huang, Lindamood, & Hansen, 1988; Määttä et al., 2007; Piispanen et al., 2009). In order to decrease product loss or to prevent interruptions of industrial waffle baking, a good‐quality waffle is characterized by high stability, that is, it is not torn during the needle take off, shows no holes or other irregularities, has an even color distribution, and does not stick to the baking plate during take‐off. Waffles that are too soft or crumbly tend to tear, even if they do not stick to the baking plate.

Sticking of waffles is greatly influenced not only by baking plate material, release agent, baking temperature and time, room temperature, and humidity, but also by the batter ingredients like type of flour or starch, sugar, fat, emulsifiers, or leavening agents (Ashokkumar, Adler‐Nissen, & Møller, 2012; Sadd, Hamlet, & Liang, 2008; Tiefenbacher & Dobrovics, 1999). In this study, the influence of the addition of starch from different sources to wheat flour as well as sugar and sugar substitutes were investigated. Starch and flour type greatly influence texture of the waffle as in any other bakery product (Huang, Lindamood, & Hansen, 1990; Maghaydah, Abdul‐hussain, Ajo, Tawalbeh, & Elsahoryi, 2013; Onyango, Mutungi, Unbehend, & Lindhauer, 2011; Pérez, Matta, Osella, de la Torre, & Sánchez, 2013) and thus highly influences waffle stability. For waffles, wheat flour of fine granulation, low to medium protein content, and low water absorption capacity should be used. In contrast to bread flour, waffle quality cannot be predicted by classical rheological parameters like farinogramm or extensogramm. Starch in flour swells during mixing and gelatinizes during baking. Proteins build up a gluten network, which provides mechanical stability, but can make a batter unusable if a too strong network is formed, which increases batter viscosity. Important is thus the mixing step of the waffle batter, for example, high shear forces or high batter temperatures should be prevented (Tiefenbacher & Dobrovics, 1999; Tiefenbacher & Wrigley, 2003). Another possibility to dilute the amount of gluten is to add starch. Lupine and rice flour, corn, tapioca, potato, and wheat starch can be used for this aim to compensate high gluten content. They can increase the batter solids and final waffle stability, but they give also a drier structure and shorten the shelf life of waffles due to increased retrogradation (Onyango et al., 2011; Tiefenbacher, 2009). Sugar and sugar substitutes are known to influence browning behavior due to Maillard reaction (Ameur, Mathieu, Lalanne, Trystram, & Birlouez‐Aragon, 2007; Mohd Jusoh, Chin, Yusof, & Abdul Rahman, 2009; Pareyt et al., 2009; Purlis, 2010), which has effects on sticking behavior of waffles. Browning is intensified with increased sugar amount and increased pH, for example, by addition of sodium bicarbonate or ammonium bicarbonate. Additionally, dissolved sugar increases the surface tension, which results in a more liquid batter, and it can reduce starch swelling during mixing, thus change the amount of required water is necessary (Ameur et al., 2007; Purlis, 2010; Tiefenbacher, 2009). Sugar granulation is very important because it influences solubility in batter and finally waffle texture. Replacing sucrose by sugar substitutes changes batter characteristic and further process parameters. Glycerine has a similar sweetness to sucrose, but shows no browning reaction as well as sorbitol, which additionally is pH and heat stable.

The objective of this study was to analyze the influence of batter ingredients on the quality (stability) and sticking behavior of waffles. Up to now, there is no detailed scientific knowledge available on these phenomena, as waffles are highly under‐researched in general. Analyzed ingredients for this part of the study were as follows; first, the effect of starches from different sources, in detail the partial replacement (30%) of wheat flour by either lupine flour, rice flour, or potato starch, and second, the effect of sugar source and sugar substitutes (crystal sugar, powdered sugar, sorbitol, and glycerine) on the quality (stability) of the waffle and on its release from the baking plates. All waffle trials were performed on pilot scale using equipment that is usually used in industrial baking.

## 2 Materials and Methods

### 2.1 Materials

The following flour and starch ingredients were used for this study: wheat flour W480 (type “Allerfeinst”, Good Mills GmbH, Schwechat, Austria; protein content 11% dm, starch content 72% dm), toasted lupine flour (Frank Food Products, Twello, NL; protein content 39% dm, starch content 20% dm), rice flour (Rickmers Reismühle, Bremen, Germany; protein content 7.3% dm, starch content 76% dm), potato starch (Agrana GmbH, Gmünd, Austria; protein content 0.5% dm, starch content 80% dm). Rapid‐visco‐analyser (RVA 4500, Perten Instruments, Macquarie Park, Australia) viscosity profiles were determined for these flour and starches. Peak/final viscosities for wheat flour were 1496/1740, for potato starch 10961 (highest)/4199, for rice flour 2942/5581 (highest), and for lupine flour 69/23 (lowest for both). Further ingredients were eggs pasteurized (Landgold Fresh GmbH, Wien, Austria), tap water (12°dH, Leobendorf, Austria), sorbitol syrup (Sorbitex Ltd., Cincinnati, USA), glycerine syrup (type 1.23, Neuber′s Enkel, Vienna, Austria), sugar (type crystal, Agrana GmbH, Tulln, Austria), skimmed milk powder (DMK GmbH, Zeven, Germany), sodium bicarbonate (Kotányi GmbH, Wolkersdorf, Austria), citric acid (Anna Gold GmbH, Vienna, Austria), a mixture of three different emulsifiers: sodium acid pyrophosphate (=SAPP40, Co. Levall, Antwerp, Belgium), monoglyceride (“Colco mono”, Co. Aromatic, Stockholm, Sweden), and lecithin (from soy, liquid, “Lecisoya” F60IP, Werba GmbH, Vienna, Austria) and rapeseed oil (100% refined, Ölwert GmbH, Langenlois, Austria). “Bandex” (Co. Aromatic, Stockholm, Sweden) was applied as release agent on the baking plates. All raw materials were stored at a temperature of 8–10°C and taken out from the cooling room 15 min prior to batter preparation.

### 2.2 Experimental design

The principal waffle recipe and its variations are given in Table 1. There is no standard recipe for fresh egg waffle. The recipe used for this study was based on a typical industrial‐scale waffle recipe as used and applied by CFT Haas Convenience Food Equipment GmbH, Leobendorf, Austria. The used ingredients and their amounts were standardized in pre‐trials (results not presented here).

**Table 1:** Experimental design—recipes of waffles investigated

Ingredients	Mixing sequence	Control [%]	Starch test^b^	Glycerine test	Sorbitol test
Wheat flour W480	3	22.40	15.68	22.40	22.40
Starch	3	0	6.72	0	0
Water 12°dH	1	7.96	7.96	7.96	7.96
Egg, fresh	1	24.89	24.89	24.89	24.89
Sorbitol syrup	1	4.98	4.98	7.47	0
Glycerine syrup	1	2.49	2.49	0	7.47
Sugar (crystal)^a^	2	15.93	15.93	15.93	15.93
Skimmed milk powder	2	1.00	1.00	1.00	1.00
Citric acid	2	0.05	0.05	0.05	0.05
Leavening Agent 1: sodium bicarbonate	2	0.29	0.29	0.29	0.29
Leavening Agent 2: SAPP40	3	0.40	0.40	0.40	0.40
Emulsifier 1: monoglyceride ColcoM	3	0.25	0.25	0.25	0.25
Emulsifier 2: lecithin liquid	4	0.50	0.50	0.50	0.50
Rapeseed oil	4	18.86	18.86	18.86	18.86
Sum	–	100.00	100.00	100.00	100.00

a in the trial with powdered sugar the full amount of crystal sugar was replaced by powdered sugar.

b Starch: lupine starch or rice flour or potato starch.

In order to study the effect of different starches, 30% of the wheat flour was replaced by lupine flour, rice flour, or potato starch. The effect of sugar alcohols was studied by either adding the whole amount of sugar alcohols as glycerine or as sorbitol in comparison to their combined addition (control recipe). Additionally, the effect of sugar granulation was studied by either adding crystal sugar or powdered sugar (see Table 1).

All these waffles were produced to best simulate industrial waffle production, using the same batter mixing machines, as well as baking plates (see section 2.4). From each recipe, 30 waffles were baked and evaluated, in order to obtain reliable results.

### 2.3 Preparation of waffle batter

Preparation of batter was performed using a dissolver stirrer (IKA‐Werke GmbH & CO. KG, Staufen, Germany) at medium speed of 800 rpm according to following common mixing sequence steps for waffles: step 1: Water and liquids (eggs, sorbitol, glycerine)—1 min; step 2: water soluble powders (sugar, skimmed milk powder, citric acid, first leavening agent)—3 to 4 min; step 3: flour, starches, and pastes (flour, starch, monoglyceride, eventually second leavening agent)—3 to 4 min; step 4: fat and lecithin prewarmed to 40°C—1 to 2 min. The mixing of the waffle batter in this sequence is important to avoid too high shearing of the batter, which would result in increased gluten development, which is not desired for waffle production. The mixed batter was allowed to rest for 15–30 min prior to baking. Batter preparation was kept constant for all recipes. All batters were monitored for pH value, temperature, density, and viscosity, in order to detect eventual influences of the varied recipe parameters.

### 2.4 Baking process for waffle production

Before baking, the baking tong “Turtle” (ductile iron baking plates, FHW Haas, Leobendorf, Austria) was preheated up to 140°C (bottom) and 145°C (top baking plate). Temperature of baking plates was monitored during the trials and found to be stable within a range of ±5°C. Before first batter disposition, a constant amount of the release agent (Bandex) was spread on both baking plates. For baking, a constant amount of 15 ml batter was deposited on the bottom baking plate, the baking plate was closed and the baking process started immediately (110 s). Then, the tong was opened and the waffle removed from the baking tong using a self‐prepared vertical needle take‐off (CFT Haas Convenience Food Equipment GmbH, Leobendorf, Austria), which is the usual process in industrial‐scale waffle production.

After a cooling period of 30 min (time for moisture equilibration within the waffle), the waffle was characterized for weight, color, color spots, moisture content, and water activity as described in the following section. Additionally, after baking of all 30 waffles and complete cooling, the baking plate was characterized visually by a microscope and for surface roughness by a surface roughness analyser (Hommel Tester T100, Hommel Seitz, Viernheim, Germany) to ensure that the baking plate surface has not changed by the baking or cleaning process, which would have an influence on sticking behavior of the waffle. During this study, surface roughness was found to be constant. Between different recipes, cleaning was carried out by dry ice (frozen CO_2_ pellets) in order to provide the same starting conditions for all recipes. In case a recipe produced many sticking waffles, an in‐between cleaning step had to be done by brushing and applying new release agent. In the results section, information is given for which recipes this step had to be applied.

### 2.5 Characterization of batter parameters (pH value, temperature, batter density, and viscosity)

The pH value of each batter was measured by a pH meter (Testo GmbH, Vienna, Austria). Optimum pH value for waffle batter lies in the range of pH 5.5 to pH 7.0. Density measurement was performed by filling a 100 ml cup with batter and recording its weight (g/100 ml). Density measurement of batter is a method to describe the aeration of a batter, the more a batter is aerated (“fluffy” batter), the lower is its density. Viscosity was measured by the so‐called flow‐cup method, which is best suitable to measure viscosity of waffle batters. The time the batter needed to flow through a 100 ml flow cup (Frikmar GmbH, Laatzen, Germany) with a 10‐mm‐diameter hole was recorded. An optimum viscosity value for waffles should be below 300 s. All these measurements were performed in triplicate and given as mean value.

### 2.6 Characterization of waffles (baking loss, moisture content, water activity, color, and color distribution)

Baking loss gives information on the moisture loss during baking and was calculated for all 30 waffles according to following formula: *Bakingloss*[%] = 1 ‐ ( *weight waffle*[*g*]/ *weight batter*[*g*])×100

For determination of the moisture content, the waffles were milled using a grinder (Co. DeLonghi, KG40, Neu‐Isenburg, Germany) and then dried by an IR dryer (MA35, Co. Satorius, Göttingen, Germany). Water activity of the waffles was measured by a water activity analyser (Labmaster‐aw, Novasin, Prague, Czech Republic). Both were determined in triplicate. Color (L*‐, a*‐, b*‐values) of the waffles was measured at three points of each waffle (right top, center, left bottom) (Colorimeter Baking Meter BC‐10, Konika Minolta, Langenhagen, Germany). In total, nine values for each recipe were used to determine the mean value. Color distribution (homogenous vs. spotted color distribution) of each of the 30 waffles were rated visually to give a rough qualitative overview in addition to the determined L*‐, a*‐, b*‐values.

### 2.7 Determination of adhesion force and number of sticking waffles

**Figure 1:**
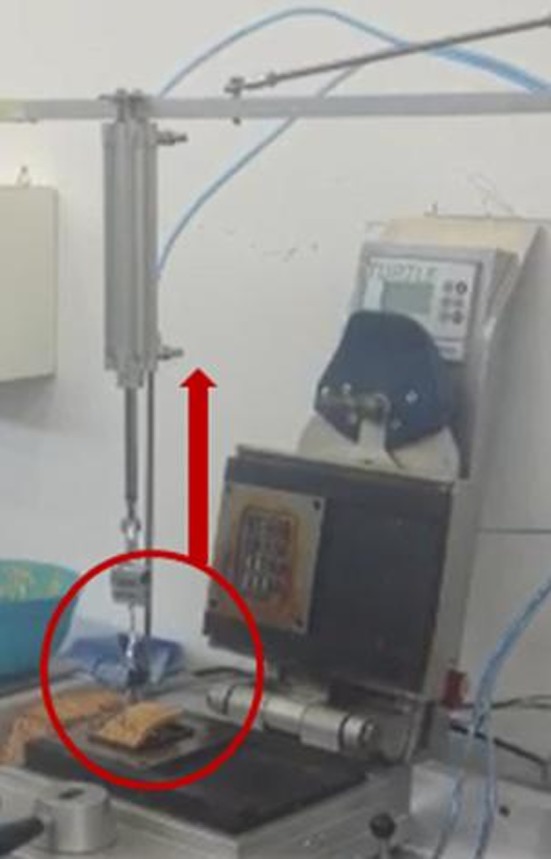
Equipment for determination of waffle adhesion force

On industrial scale, usually waffles are taken off from the baking plates vertically by needles. In order to best simulate this process, a so‐called needle take‐off was simulated by an in‐house construction for pilot‐scale production, which allowed measuring the force required to take off the waffle (see Fig. 1). After opening the baking tong, the arm with the needles was moved above the waffle, the needles were put into the waffle always at the same position, the scale was tared and then the needles were pulled up vertically by pneumatic force. The pneumatic pressure and the angle of the take‐off arm was set constant and proofed by a barometer. A hanging scale (HS 300± 0.1 g, Co. Dipse, Oldenburg, Germany) recorded the weight required to take‐off the waffle from the baking tong, which was used to calculate the adhesion force by following formula:

$$\text{Adhesion force}\left[N=kg\times m/s^{2}\right]=\text{Waffle take‐off weight}\left[g\right]/1000\times 9.81\left[m/s^{2}\right].$$

Depending on the intensity of adhesion, the weight of the taken off waffle increased.

Number of sticking waffles was counted for all 30 waffles produced for one recipe and given as %. Torn waffles (not or only half taken off) were considered as sticking waffles.

### 2.8 Statistical evaluation

Results of waffles were evaluated statistically using Statgraphics Centurion XVI, Statpoint Technologies, Inc., Warrenton, USA) by one‐factor analysis of variance (ANOVA) and Spearman rank correlation between each pair of variables. A *p*‐value below .05 was considered as significant. Mean values were compared by Fisher's least significant difference (LSD) procedure.

## 3 Results and Discussion

**Figure 2:**
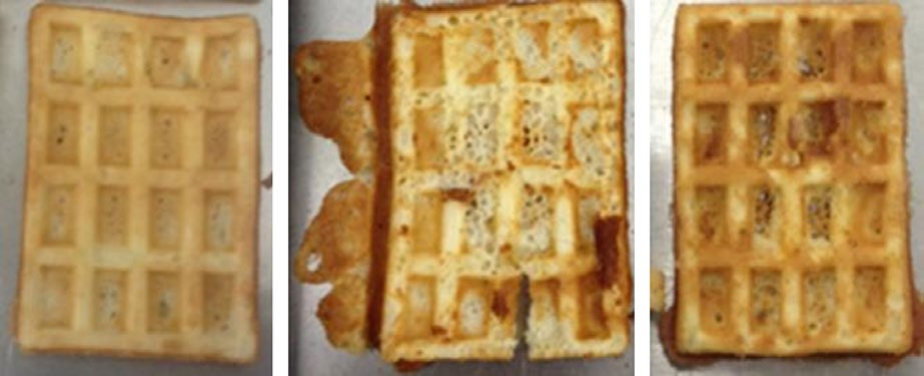
Examples for waffles with good and bad quality (from left to right: wheat flour and glycerine (homogenous color distribution and totally filled waffle), rice flour (spotted and broken waffle), wheat flour (spotted, holes, crumble texture)

All batter parameters determined are summarized in Table 2, waffle parameters in Tables 3 and 4 and Figure 2. Correlation analyses of the determined parameters are presented in Table 5.

**Table 2:** Batter characterization: pH, temperature, density, and viscosity (*n* = 3)

Batter characterization	pH	Temperature[°C]	Density[g/L]	Viscosity[s]
Effect of flour and starch				
Wheat flour ISTD	6.36 ± 0.01^d^	26.2 ± 0.2^b^	92.47 ± 0.42^b^	120 ± 5^c^
Lupine flour	6.13 ± 0.02^a^	22.6 ± 0.1^a^	86.29 ± 1.41^a^	291 ± 10^d^
Rice flour	6.31 ± 0.03^c^	22.8 ± 0.2^a^	86.089 ± 1.16^a^	65 ± 7^b^
Potato starch	6.22 ± 0.01^b^	21.0 ± 0.1^a^	92.94 ± 1.29^b^	36 ± 1^a^
*p‐value*	.0000	.0000	.0001	.0000
Effect of sugar and substitutes				
Sorbitol syrup	6.51 ± 0.01^c^	23.0 ± 0.1^b^	89.25 ± 1.33^a^	114 ± 4^c^
Glycerine syrup	6.41 ± 0.02^b^	23.8 ± 0.2^c^	95.57 ± 0.98^c^	64 ± 2^a^
Sugar powdered	6.34 ± 0.01^a^	21.7 ± 0.2^a^	92.49 ± 1.44^b^	84 ± 6^b^
Sugar crystal ISTD	6.36 ± 0.01^a^	26.2 ± 0.2^d^	92.47 ± 0.42^b^	120 ± 5^c^
*p*‐value	.0000	.0000	.0010	.0000

Note

Different superscript letters in the same column denote significant differences (p<0.05)

**Table 3:** Waffle characterization: color values (L*‐, a*‐ and b*‐values; *n* = 30)

	L*	a*	b*
Effect of flour and starch			
Wheat flour ISTD	46.31 ± 6.61^ab^	16.58 ± 2.93^a^	29.94 ± 2.56^a^
Lupine flour	45.46 ± 5.11^a^	18.83 ± 2.41^b^	32.37 ± 3.38^b^
Rice flour	49.25 ± 6.83^b^	16.28 ± 3.73^a^	32.63 ± 2.25^b^
Potato starch	47.24 ± 6.18^ab^	16.19 ± 2.50^a^	30.88 ± 2.24^a^
*p‐value*	.1096	.0070	.0003
Effect of sugar and substitutes			
Sorbitol syrup	59.56 ± 7.37^b^	11.78 ± 3.93^b^	31.92 ± 2.57^b^
Glycerine syrup	66.20 ± 5.08^c^	8.80 ± 2.77^a^	33.50 ± 2.75^c^
Sugar powdered	63.74 ± 3.80^c^	10.87 ± 2.66^b^	33.56 ± 2.43^c^
Sugar crystal ISTD	46.31 ± 6.61^a^	16.58 ± 2.93^c^	29.94 ± 2.56^a^
*p‐value*	.0000	.0000	.0000

Note

Different superscript letters in the same column denote significant differences (p<0.05)

**Table 4:** Waffle characterization: baking loss, moisture content, aw‐value, take‐off force, and sticking of waffles

	Baking loss (*n* = 30)[%]	Moisture [%] (*n* = 3)	aW (*n* = 3)	Take‐off Force (*n* = 30)[N]	Sticking (*n* = 1) [%]
Effect of flour and starch					
Wheat flour ISTD	25.41 ± 3.31^ab^	13.31 ± 0.94^a^	0.68 ± 0.03^a^	0.044 ± 0.015^b^	23.3 ± 43.0^a^
Lupine flour	24.47 ± 2.83^a^	14.38 ± 1.22^a^	0.70 ± 0.02^a^	0.032 ± 0.008^a^	6.7 ± 25.4^a^
Rice flour^a^	25.13 ± 2.62^ab^	14.11 ± 0.68^a^	0.70 ± 0.01^a^	0.031 ± 0.012^a^	46.7 ± 50.7^b^
Potato starch	26.50 ± 2.17^b^	13.90 ± 0.77^a^	0.69 ± 0.03^a^	0.027 ± 0.005^a^	6.7 ± 25.4^a^
p‐value*	.0425	.5660	.7919	.0000	.0001
Effect of sugar and substitutes					
Sorbitol syrup^a^	20.93 ± 3.87^a^	14.22 ± 1.87^a^	0.71 ± 0.02^b^	0.025 ± 0.008^b^	26.7 ± 45.0^b^
Glycerine syrup	22.65 ± 3.46^ab^	14.08^a^	0.63^a^	0.020 ± 0.004^a^	0.0 ± 0.0^a^
Sugar powdered	23.44 ± 4.27^b^	13.09 ± 0.40^a^	0.64 ± 0.02^a^	0.029 ± 0.007^b^	20.0 ± 40.7^b^
Sugar crystal ISTD	25.41 ± 3.31^c^	13.31 ± 0.94^a^	0.68 ± 0.03^ab^	0.044 ± 0.015^c^	23.3 ± 43.0^b^
*p‐value*	.0001	.6766	.0286	.0000	.0289

a These recipes required an in‐between cleaning step (brushing and additional application of releasing agent). Different superscript letters in the same column denote significant differences (p<0.05).

**Table 5:** Correlation analyses of determined batter and waffle parameters

	Temperature	Density	Viscosity	Moisture	aW‐value	L*	a*	b*	Baking loss	Force	Stickingwaffles
pH	0.5741^a^	0.3554	0.0033	−0.154	−0.0478	0.5057^a^	−0.6942^a^	0.2599	−0.5924^a^	−0.328	0.1364
Temperature		0.3823	0.5124^a^	−0.1406	−0.0636	0.1651	−0.2759	0.0514	−0.4559^a^	0.0182	−0.2273
Density			−0.3935	−0.1982	−0.4852^a^	0.5273^a^	−0.5705^a^	0.258	−0.0526	−0.4283	0.2636
Viscosity				−0.1596	0.1341	−0.2688	0.3054	−0.0864	−0.1994	0.4108	−0.036
Moisture					0.6387^a^	−0.1105	0.2564	−0.0852	0.007	−0.0246	−0.1
aW						−0.322	0.2958	−0.4009	0.0062	0.1135	−0.3545
L*							−0.8778^a^	0.5405^a^	−0.6452^a^	−0.5475^a^	0.5946
a*								−0.2454^a^	0.534^a^	0.5416^a^	−0.5946
b*									−0.4246^a^	−0.2341^a^	0.8469^a^
Baking loss										0.3582^a^	0.018
force											0.3424

a Significant at a *p*‐value below .05.

### 3.1 Effect of ingredients on batter parameters

In pre‐trials (results not shown here), the control batter recipe was composed in a way that pH, density, and viscosity were within a recommended range for waffle batters. These recommended ranges are well experienced for industrial‐scale waffle products by CFT‐Haas GmbH (Leobendorf, Austria). Based on this recipe, the effect of varied ingredients was investigated and it was monitored if and in what range they changed these values. Severe changes of these parameters would already indicate their unsuitability for waffle production.

The pH value of the batters ranged from 6.1 to 6.5 and was not much influenced by the selected recipe components. From industrial trials (own experience, no data presented), it is known that high pH‐values of batter can lead to increased sticking of the waffles. This is explained by the fact that at higher pH‐values the browning reaction is intensified, which increases the amount of batter residues on the baking plates over time. In this performed trials, this phenomenon could not be detected.

Batter temperature ranged from 21.0 to 26.6°C, which was in the recommended range for waffle production. If the batter temperature is too high, the batter tends to form clumps, which leads to nonreproducible and too high batter deposit amounts and which could increase the number of sticking waffles.

Density and viscosity of batters are two parameters that have important effects on the fluidity properties of batters and later on waffle quality. Density of a waffle batter should be around 80–95 g/100 ml in order to show good fluidity behavior to fill the whole baking plate after application. A highly liquid dough causes higher waste of dough. Viscosity in liquids is usually influenced by density (the higher density the higher viscosity), but in batters, the effect of aeration on viscosity is much more relevant. The higher the aeration, the higher the batter viscosity, in practical terms spoken the batter gets stiffer (foam structure). Too stiff doughs again reduce or limit their fluidability and spreadability. Within a recipe, density and viscosity are negatively correlated and are influenced by aeration only—the higher the aeration, the lower the density and the higher viscosity. But density and viscosity (or foaming properties) are also strongly influenced by recipe ingredients and are thus only correlated within one recipe but not across different recipes. Some recipes show high density but low foamability after aeration or reverse. Principally, better aerated batters lead to softer waffles, but for sufficient spreadability, viscosity should remain below 5 min (as determined by the flow cup method). Softer waffles might have an influence on waffle stability, but not necessarily on sticking behavior.

Density and viscosity were significantly influenced by the addition of starch and sugar substitutes. Replacement of wheat flour by potato starch did not change density compared to wheat flour but decreased viscosity. Replacement by lupine flour decreased density, and increased viscosity strongly. This is most likely the result of the lupine protein, which is known to have good foaming ability (Maghaydah et al., 2013; Pareyt et al., 2009). Rice flour replacement decreased density and viscosity. Sole glycerine syrup addition showed higher density than sole sorbitol syrup or their combination, but sole glycerine syrup decreased viscosity. Between crystal and powdered sugar, there was no difference in density or viscosity. Some significant correlations between batter parameters and waffle parameters could be found, for example, positive correlation between pH‐ and L‐value, negative correlations between pH‐ and a‐value, or density and aw‐value, but all association values were rather low, thus only indicating weak relationships. All recipes showed values for density and viscosity that were in an acceptable range for waffle batters and thus no significant effect of density or viscosity on sticking behavior or adhering force was detected. To conclude these results shortly, principally none of the selected ingredients had a negative influence on waffle batter properties. From this point of view, they were principally all suitable for waffle production.

### 3.2 Effect of ingredients on general waffle quality

The investigated starches, sugar sources, or sugar substitutes influenced the appearance of the waffles. Visually, it could be observed that potato starch addition resulted in waffles of high stability with more homogenous color distribution than the waffles produced with wheat flour only or with replacement by lupine or rice flour. Glycerine syrup performed better than sorbitol syrup and crystal sugar better than powdered sugar with respect to stability and color distribution. During the production of the waffles with rice flour and the ones with sorbitol, in‐between cleaning (by hand‐brushing) and additional application of release agent was necessary. In comparison to the other flours used in this study, potato starch contained the highest amount of starch (80%) and showed the highest RVA values, which provides more elastic properties of the starch gelatinization itself compared to other tested starches and flours. Potato starch has an amorphous and more ordered structure (Stasiak, Rusinek, Molenda, Fornal, & Błaszczak, 2011). Lupine flour contained less starch (20%) but a protein that is known to have good foaming properties, thus it resulted in softer end‐products, which might have been a disadvantage in waffle production. In a study on the effect of lupine flour on baking characteristics of gluten‐free cookies, Maghaydah et al. (2013) demonstrated that addition of starch improved waffle stability in general and addition of lupine flour softened the texture.

Color is an important analysis for the bakery industry. Color determination is not directly influencing sticking behavior, but it gives some information on the state of the waffles. Color of the waffles was influenced by starch addition. Lupine flour resulted in darker waffles compared to rice flour. Lupine flour addition lead to higher a*‐ and b*‐ values. Regarding sugar components, waffles with glycerine syrup addition were less red, but more yellow compared to those with sorbitol syrup, and powdered sugar made the waffles less red and more yellow (lower a*‐ and higher b*‐value) in comparison to crystal sugar. Obviously, powdered sugar decreased the production of Maillard products. It is known that darker color of waffles is usually caused by higher sugar amounts (increasing Maillard reaction) (Tiefenbacher, 2003; Ronda, Oliete, Gómez, Caballero, & Pando, 2011), but differences in color properties by the incorporation of crystalline or powdered sucrose are not known from other studies yet. Own experiences showed that crystalline and powdered sucrose show different sticking characteristics, which is assumed to occur due to the different dissolving behavior during batter preparation. Differences in color properties can be caused by protein content and type of protein, as Shevkani, Kaur, Kumar, and Singh (2015) has shown for muffins. Glycerine and sorbitol can influence the protein structure within a batter or dough. The water‐retaining properties of sorbitol and glycerine lead to an increased moisture content of the waffles, which reduced the required take‐off force (Demuth et al. 2015; Nezzal, Aerts, Verspaille, Henderickx, & Redl, 2009; Willart et al., 2005). Sugar sources can influence moisture content or water activity differently (Pareyt et al., 2009).

The aim of measuring moisture content was to follow waffle quality (Seog, Kim, & Lee, 2008). No differences were found between the starches or flours, but the different sugar sources influenced water activity. Glycerine syrup caused lower water activity in comparison to sorbitol syrup. Glycerine and sorbitol syrup act as moisture‐binding agents, thus they principally can decrease water activity at same moisture content. There was no clear relationship of moisture or water activity to sticking behavior.

Baking loss was significantly influenced by starch and sugar addition; it was higher for potato starch compared to lupine flour, slightly higher for glycerine compared to sorbitol syrup (not significant) and for crystal sugar compared to powdered sugar, but differences were very low. Also, other researchers (Ashokkumar & Adler‐Nissen, 2011; Ashokkumar et al., 2012) found that sticking of pancakes was influenced by moisture loss of the products. Color was significantly correlated with a lower baking loss (the darker, more red and less yellow the waffles the lower was baking loss), but association was low. Most likely the darkening effect occurred due to an intensified baking process, as other studies showed similarly for cakes or cookies (Ferng, Liou, Yeh, & Chen, 2016; Hesso et al., 2015; Pareyt et al., 2009; Purlis, 2010).

### 3.3 Effect of ingredients on sticking behavior of waffles

Sticking behavior was determined by calculating the number of sticking waffles (% of 30 waffles) and measuring the take‐off force. A waffle that was torn during take‐off due to partially sticking increased the take‐off weight.

The investigated ingredients showed significant influences on sticking behavior and take‐off force. Within the starch group, rice flour caused the highest percentage of sticking waffles (46%), although take‐off force was in the same range as lupine flour and potato starch and even lower compared to wheat starch (all 6.7%). As described before, rice flour resulted in very soft and inhomogeneous waffles, so almost every second one could not be removed, but the ones that were stable enough to be taken off, required only little force. Rice flour is known to give cakes a firmer structure, which was assumed to be due to its lower amylose content. This might also explain the dry, crumbly texture of the waffles, compared to the spongy‐chewy texture of the waffles with potato starch. Glycerine syrup was again superior to sorbitol syrup or its combined addition, as well as crystal sugar compared to powdered sugar. They required less take‐off force. Take‐off force was significantly correlated with L*‐ and b*‐value (negative, but weak association) and positive to a*‐value. Sticking behavior showed strong associations to b*‐value (positive correlation) and to a*‐value (negative correlation). The lower the L*‐ and b*‐value and the higher the a*‐value (i.e., the waffle was darker, less yellow and more red), the higher was the take‐off force and the lower was the sticking behavior of the waffle. A waffle that had a regular color (rather light, less red but more yellow, indicating also less spots) was more stable and less sticking and required significant lower take‐off forces. An increase in baking loss seemed to increase take‐off force, but the positive association was only weak.

From these determinations, it could be concluded that in order to obtain high waffle stability and lowest sticking tendency, baking loss should not be too high, batter temperature should be within an optimum range (20–28°C), color should be very regular and not too dark. To obtain these effects, it was shown that an increase in pH of the batter and addition of ingredients with increased water holding capacity to limit baking loss can play a positive role.

Also, in other studies, sticking or adhering behavior of food was influenced by adherence or cohesive forces and by stability of the sample. Liu, Christian, Zhang, and Fryer (2002) investigated the take‐off for various foods like tomato or whey protein and found different adhesive forces of different foods, which suggests that different ingredients have an effect on adhesion or sticking behavior. Additionally, they observed that removing the food from a heated plate required increased force when the food broke during this procedure.

The results of this study have shown that take‐off force and sticking of waffles were depending on recipe ingredients of flours, starches, sugar, and sugar substitutes. They influenced waffle stability. An unstable waffle can break and stick due to insufficient waffle structure or because of actual sticking and burning reactions. Increased adhesion force might lead to increased sticking behavior over long‐term baking processes.

## 4 Conclusion

In this study, effects of different starch sources and sugar components could be demonstrated: waffles with increased stability and texture (elastic, not too dry, and crumbly) were those that showed the least number of sticking waffles, thus the main aim of batter ingredients was to improve waffle quality (stability, texture) in order to decrease sticking and thus food waste.

Within the selected starches, potato starch demonstrated the highest effects on increasing waffle stability and releasing properties. Sticking number of waffles made from potato starch were comparable to waffles made from wheat flour and lupine flour, but the waffles from wheat flour and lupine flour were softer and crumblier. Take‐off force was higher for these waffles, so in long‐term trials they are more at risk to develop increased sticking behavior. For all starches investigated, rice flour addition performed worst, with almost 50% of sticking waffles. Most of these waffles were broken during take‐off, due to their crumbly texture. Within the sugar components, glycerine was better suitable than sorbitol and crystal sugar was superior compared to powdered sugar. Glycerine in particular provided better texture, color distribution, and release characteristics of the waffles than sorbitol. For optimum waffle quality and enhanced production, the obtained results suggest developing waffle batters with a balanced addition of potato starch to wheat flour, to add glycerine instead of sorbitol and to incorporate ingredients with increased water holding capacity. A moderate baking process should be applied. Overall, the main aim was to achieve a stable waffle texture, which supports the waffle take‐off during industrial baking.

## Funding Information

No funding information provided.

## Conflict of Interest

None declared.
